# In Vitro Cocktail Effects of PCB-DL (PCB118) and Bulky PCB (PCB153) with BaP on Adipogenesis and on Expression of Genes Involved in the Establishment of a Pro-Inflammatory State

**DOI:** 10.3390/ijms19030841

**Published:** 2018-03-13

**Authors:** Phealay May, Patricia Bremond, Christophe Sauzet, Philippe Piccerelle, Frédérique Grimaldi, Serge Champion, Pierre-Henri Villard

**Affiliations:** Aix Marseille Univ, Univ Avignon, CNRS, IRD, IMBE, Faculté de Pharmacie 27 Bd Jean Moulin, 13385 Marseille CEDEX 5, France; phealaymay@gmail.com (P.M.); patriciabremond@live.fr (P.B.); christophe.sauzet@univ-amu.fr (C.S.); philippe.piccerelle@univ-amu.fr (P.P.); frederique.grimaldi@univ-amu.fr (F.G.); serge.champion@univ-amu.fr (S.C.)

**Keywords:** polychlorobiphenyls (PCB), polycyclic aromatic hydrocarbons, adipocytes, cocktail effect, adipogenesis, inflammation, Ah receptor, benzo(a)pyrene

## Abstract

(1) Objective: Highlight the in vitro effects of 3T3-L1 cell exposure to polychlorinated biphenyls (PCB118 and 153) or benzo(a)pyrene (BaP) alone or as a cocktail on adipogenesis (ADG) by focusing on changes in lipid metabolism and inflammatory-related genes expression (INFG) and ADG-related genes expression (ADGG); (2) Results: Treatment from the early stage of cell differentiation by BaP alone or in combination with PCBs decreased the expression of some of the ADGG (*PPARγ* *Glut-4*, *FAS*, *Lipin-1a*, *Leptin*, and *Adiponectin*). BaP enhanced the INFG, especially *MCP-1* and *TNFα*. Co-exposure to BaP and PCB153 showed a synergistic effect on *TNFα* and *IL6* expression. Treatment with BaP and PCBs during only the maturation period up-regulated the INFG (*IL6*, *TNFα*, *CXCL-10 & MCP-1*). PCB118 alone also enhanced *TNFα*, *CXCL-10*, and *PAI-1* expression. The change in MCP-1 protein expression was in agreement with that of the gene. Finally, the BaP-induced up-regulation of the xenobiotic responsive element (XRE)-controlled luciferase activity was impaired by PCB153 but not by PCB118; (3) Conclusion: BaP and PCBs down-regulate a part of ADGG and enhance INFG. The direct regulatory effect of PCBs on both ADGG and INFG is usually rather lower than that of BaP and synergistic or antagonistic cocktail effects are clearly observed.

## 1. Introduction

The global prevalence of type 2 diabetes (T2D) is increasing rapidly, running parallel to the increase in obesity. Globally, an estimated 422 million adults were living with diabetes in 2014, compared to 108 million in 1980. The global prevalence (age-standardized) of diabetes has nearly doubled since 1980, rising from 4.7% to 8.5% in the adult population [1]. This worldwide pandemy of T2D appears only partly caused by the reduction in physical activity, the adoption of a sedentary lifestyle and the genetic backgrounds. It has become clear that environmental factors are also involved. Indeed we are exposed in our environment to numerous chemical compounds, including residues present in food. Studies assessing their detrimental effects are generally performed on only one isolated compound. Cocktail effects are poorly studied, while they can lead to additive, potentiative, or antagonist effects. Therefore, it was not possible to extrapolate the effects of chemical cocktails from data obtained with isolated compounds only.

T2D is characterized by a chronic low-grade inflammation, notably of white adipose tissue and liver. Such an insulin resistance-associated pathology has been recently referred to as ‘metaflammation’. Obesity induces an increased risk of metabolic diseases, including insulin resistance and T2D [2]. However, exposure to some environmental pollutants either in isolation or in the form of cocktails can also enhance the risk of T2D. Meta-analyses indeed provide quantitative evidence consistent with the hypothesis that environmental exposure to polychlorinated biphenyls (PCBs) is a contributing risk factor for the prevalence of T2D [3,4].

PCBs are organochlorines considered as persistent organic pollutants (POPs). They are very stable and resistant to a large range of physico-chemical environmental conditions. They are fat-soluble compounds that bioaccumulate in individuals and are bio-magnified in the food chain. They were first commercially produced in the United States in 1929 and used widely in capacitors, transformers, hydraulic fluids, heat transfer fluids, lubricants, plasticizers and as components of surface coatings and ink. There are 209 possible PCB congeners which are categorized based on structural properties such as the number of chlorine atoms in the compound. A common classification divides PCBs into dioxin-like (which are agonists of Ah receptor (AhR)) and nondioxin-like (which are agonists of constitutive androstane receptor (CAR) or pregnane X receptor (PXR)), based on their structural and toxicological similarity with the dioxin molecule [5,6]. Adverse effects of PCBs have been well documented in animal and human studies. These include carcinogenicity, reproductive impairment, neuro-developmental anomalies, immunologic deficiency, and endocrine adverse effects [7,8]. As mentioned above, PCB exposure was associated to an increased risk of T2D [3,4], but the molecular mechanisms of this detrimental effect of PCB are poorly understood. In a previous in vivo work, we have demonstrated in mice, that adipose tissue is the main target organ affected by metabolic disorders induced by PCB [9]. In this work, transcriptomic analyses highlighted the down-regulation of two genes involved in insulin sensitivity and glucose homeostasis: Glut-4 and Lipin-1. Interestingly, we failed to detect hyperglycemia in mice exposed to PCB118 or PCB153, suggesting that PCB congener alone is not sufficient to induce by itself a diabetic state.

Human exposure to PCBs occurs through food consumption, mainly fishes (especially when taken from lakes or rivers containing high levels of PCBs) and also meat. In addition, other compounds present in food can activate AhR. Among them, Polycyclic Aromatic Hydrocarbons (PAH), such as benzo(a)pyrene (BaP), are particularly of interest. They activate indeed AhR and are metabolized by *CYP1*—genes regulated by AhR pathway—into electrophilic compounds that exert mutagenic and pro-inflammatory effects [10,11,12]. Recently, urinary high levels of PAHs biomarkers were found to be positively associated with diabetes mellitus in humans [13]. Therefore, it appeared advisable to evaluate the cocktail effect of PAHs and PCBs on adipose tissue, one of the main tissue at the origin of T2D-associated inflammation.

So, the aim of the present work was to study the in vitro cocktail effects of BaP (the PAH of reference), and either PCBs-DL (PCB118), or bulky PCBs (PCB153), on adipocyte physiology, using 3T3-L1 cells as a model.

## 2. Results

### 2.1. Cytotoxicity and Treatments Procedure

**Figure 1:**
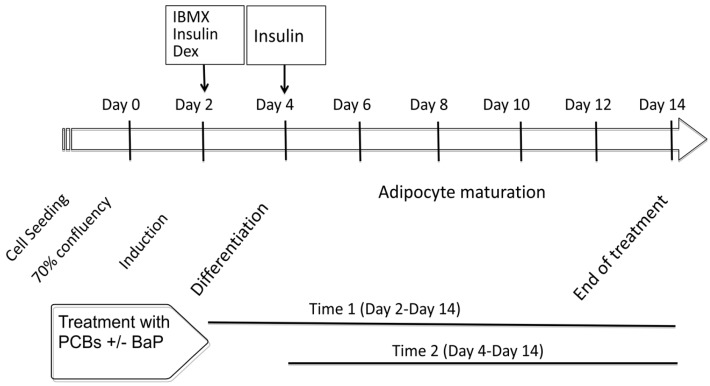
Treatment schedule of 3T3-L1 cells with benzo(a)pyrene (BaP) and/or polychlorinated biphenyls (PCBs) (PCB118 or PCB153) from the induction of differentiation (Time 1: day 2–day 14) or after differentiation (Time 2: day 4–day 14) to the maturation state (day 14). Dex: dexamethasone. IBMX: 3-isobutyl-1-methylxanthine.

**Figure 2:**
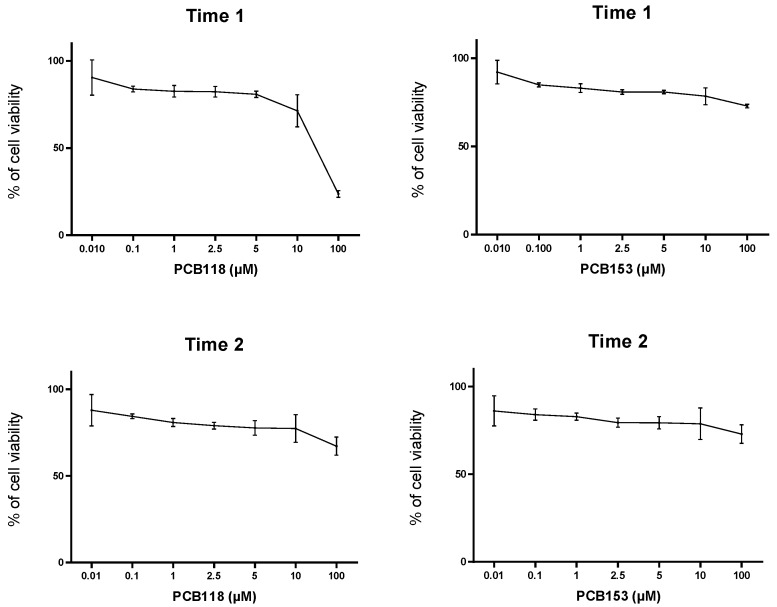
Results of MTT assays performed in “Time 1” and “Time 2” conditions after PCB118 or PCB153 exposure. 3T3-L1 cells were incubated with or without PCB118 (one or five micromolars) or PCB153 (one or five micromolars) according to the “Time 1” or “Time 2” conditions, and MTT assays were performed as described in materials and methods section.

The MTT test led us to demonstrate (see Figure 1 and Figure 2) that, in this cellular model, PCBs exhibit a low cytotoxic effect, and IC_50_ for these compounds was reached at the highest concentration studied (100 µM). Therefore, the experiments were undertaken with non-cytotoxic doses (usually one and five micromolars) compatible with the human exposures. BaP was used at the final concentration of one micromolar, since with doses above five micromolars around 10% of cell mortality was observed (Laboratory unpublished data).

As reported in Section 4, the experiments focused on the measurement of lipid formation and expression of genes involved in the adipogenesis, glucose and lipid homeostasis, inflammation and production of adipokines in response to cell treatment by organic pollutants (PCB118, PCB153 and BaP) alone or in combination at the onset of the induction process from day 0 (see schedule diagram) and during the entire differentiation period or only during the adipocyte differentiation (from D2) (Figure 1). Hereafter, these periods of treatment are named respectively “Time 1” and “Time 2”. Some control experiments were also carried out on undifferentiated cells reaching 70–80% of confluence.

### 2.2. Effects of PCB118, PCB153 and BaP on Oil Red O Staining of Adipocyte Lipids

In order to evaluate globally the effects of the studied organic pollutants on the differentiation of 3T3-L1 adipocytes, we performed measurements by spectrophotometry of lipid synthesis, using the oil red O staining method.

**Figure 3:**
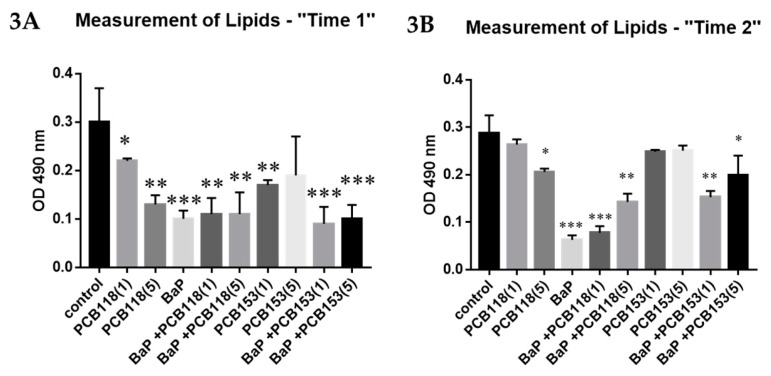
Effects of PCB118, PCB153 and BaP on “Oil Red O” staining of adipocytes ((**A**) “Time 1”; (**B**) “Time 2”). 3T3-L1 cells were incubated with or without PCB118 (one of five micromolars) or PCB153 (one or five micromolars) in the presence or not of BaP (one micromolars) according to the “Time 1” or “Time 2” conditions described in materials and methods. The measurement of cellular lipids was carried out using the “Oil Red O” staining method as described under materials and methods. Each incubation condition was carried out in quadriplicate and the experiment was repeated three times. The values are represented as mean ± SD. *n* = 4 per group. The level of significance is given by comparison of treated trials with controls: * *p* < 0.05; ** *p* < 0.001; *** *p* < 0.0001.

**Figure A1:**
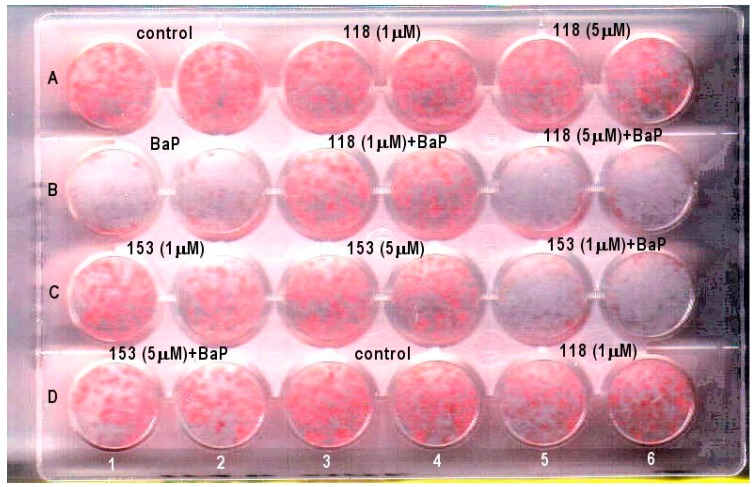
Effects of PCB118, PCB153 and BaP on “Oil Red O” staining of adipocytes in Time 1 conditions. 3T3-L1 cells were incubated with or without PCB118 (one or five micromolars) or PCB153 (one or five micromolars) in the presence or not of BaP (one micromolar) according to the “Time 1” or “Time 2” conditions described in materials and methods. The measurement of cellular lipids was carried out using the “Oil Red O” staining method as described under materials and methods.

When pollutants are used at the early phase of the differentiation process (“Time 1”), we observed (Figure 3 and Appendix A, Figure A1) that in BaP-treated group, Oil red O staining was markedly reduced. PCBs alone induced a slight decrease of lipid staining, except in experiments with PCB118 at five micromolars, in which the effects were comparable to those obtained with BaP. When PCBs were added mixed with BaP, the results were comparable to those observed with BaP alone.

Similar results are obtained in “Time 2” treatment but the effect of PCBs was slightly lower while that of BaP was stronger and the coexposure of cells to both kinds of compounds led to a reduction of the effect of BaP on lipid formation.

### 2.3. Effects of PCB118, PCB153 and BaP on CYP1A1 Gene Expression

*CYP1A1* is a well-known AhR target gene, and both compounds BaP and PCB118 are potent AhR agonists.

**Figure 4:**
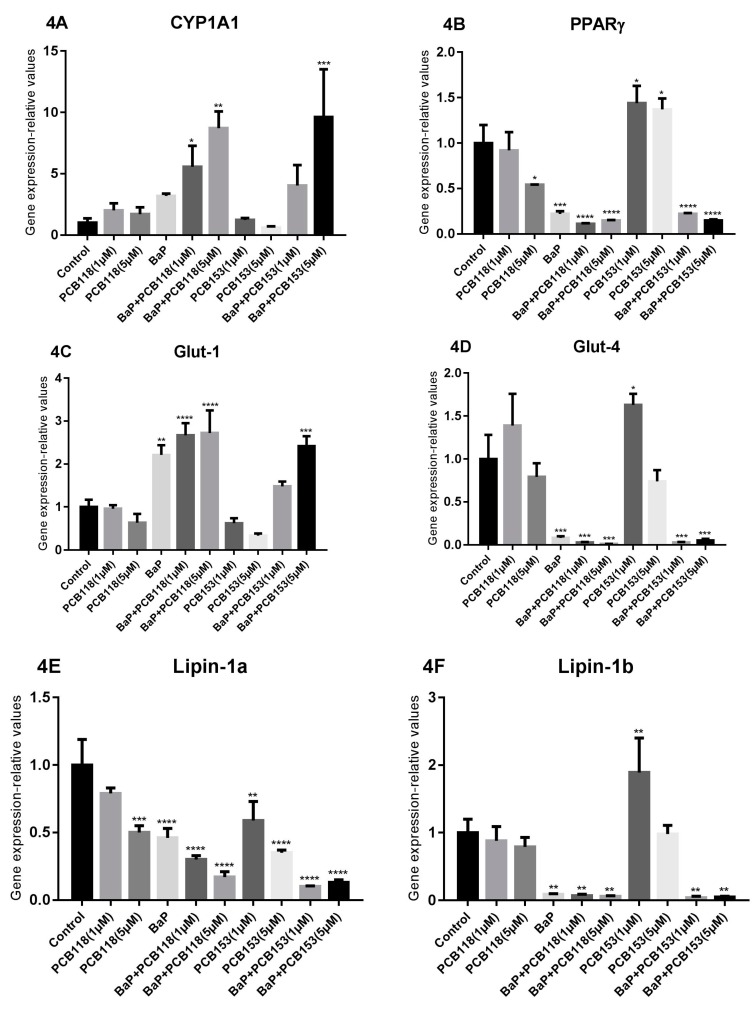

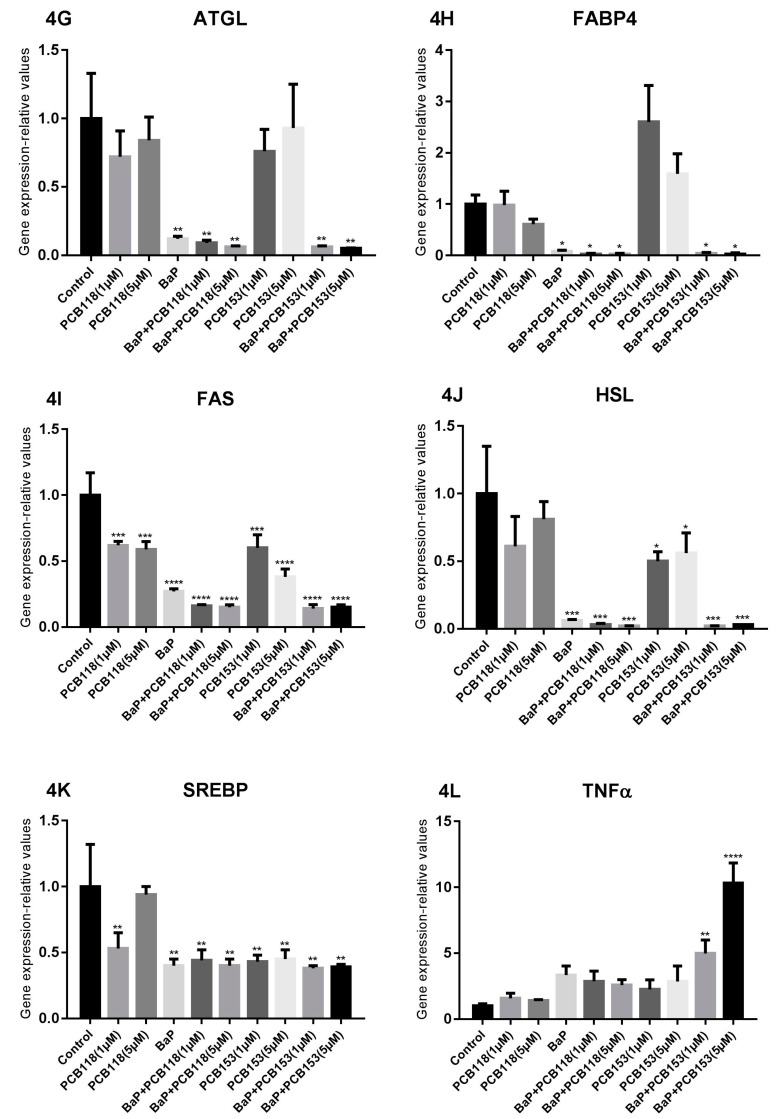

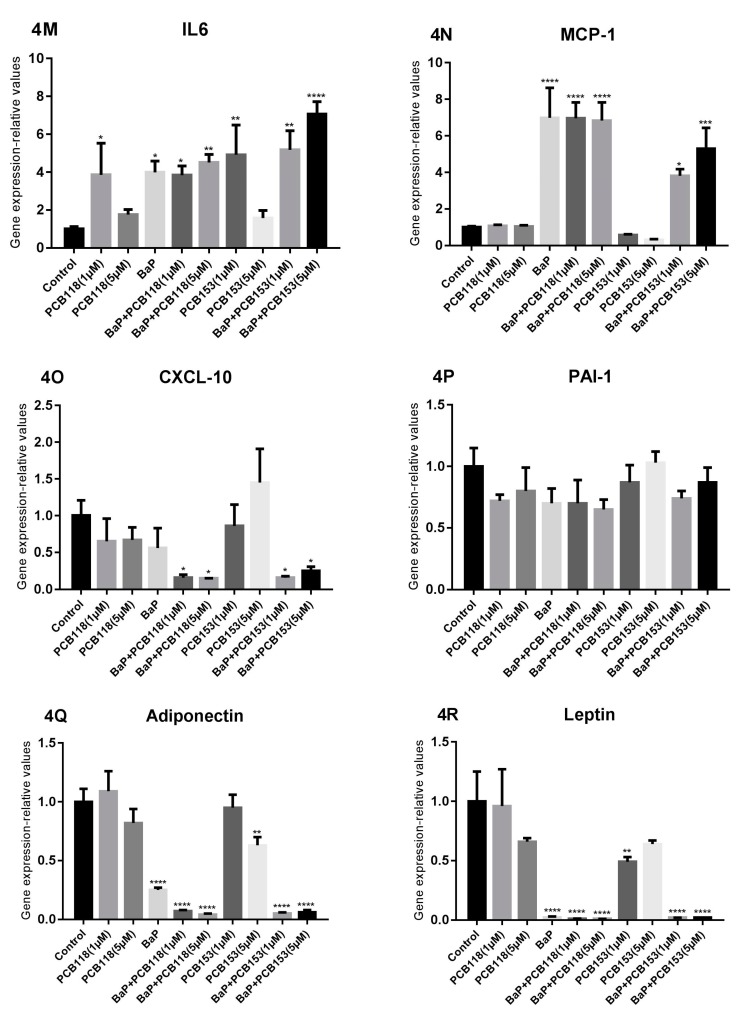
Effects of PCB118, PCB153 and BaP in “Time 1” conditions on the expression of genes related to adipocyte differentiation, glucose and lipid homeostasis, and inflammation. 3T3-L1 cells were incubated with or without PCB118 (one or five micromolars) or PCB153 (one or five micromolars) in the presence or not of BaP (one micromolar) according to the “Time 1” conditions described in materials and methods. Cells were washed twice with PBS and mRNA extraction and qRT-PCR were performed as described under materials and methods. Each incubation condition was carried out in quadriplicate and the experiment was repeated three times with similar results. The data are mean ± SD. The level of significance is given by comparison of treated trials with controls (*n* = 4 per group): * *p* < 0.05; ** *p* < 0.01; *** *p* < 0.001; **** *p* < 0.0001.

Addition of organic pollutants at the early stage of the induction process, i.e., “Time 1” led to a lower induction of *CYP1A1* expression than expected (Figure 4): a low induction following treatment of the cells with BaP and PCB118, and no induction with PCB153. However, when the cells were co-treated with BaP and PCB118, we observed a potentiation of the *CYP1A1* induction and surprisingly, such a potentiation was also observed in response to the combination of BaP with PCB153.

When pollutants were tested at “Time 2”, only BaP induced significantly the CYP1A1 expression. Surprisingly, this induction of CYP1A1 was partly repressed by a co-addition of PCB118 or PCB153.

### 2.4. Effects of PCB118, PCB153 and BaP on the Expression of Genes Related to Adipocyte Differentiation, Glucose and Lipid Homeostasis

PPARγ is generally referred as the “master regulator” of adipogenesis. This factor is both necessary and sufficient for the adipogenesis induction. The general program of fat cell differentiation is coordinated by PPARγ and members of the c/EBP family of transcription factors. c/EBPβ is induced early during the process of adipogenesis and plays a critical role in activating *PPARγ* expression. In fat cells, PPARγ, in cooperation with c/EBPα, binds and regulates the expression of most adipogenesis-related genes [14].

By adding organic pollutants at the early stage of the induction process, i.e., “Time 1” treatment, we can see (Figure 4) that BaP repressed markedly the expression of *PPARγ* gene. As generally reported in the literature, the down-regulation of *PPARγ* expression is generally associated to a decrease of the expression of genes involved in glucose and lipid homeostasis such as *Glut-4*, *Lipin-1a*, *Lipin-1b*, *ATGL*, *FABP4*, *FAS*, *HSL*, and *SREBP*. By contrast, in these same conditions an induction of *Glut-1* expression was observed.

In our experiments, in the “Time 1” treatment conditions, PCB alone caused a slight biphasic effect on *Glut-4* gene expression, which was enhanced at one micromolar and decreased at five micromolars, while Glut-1 expression was only faintly reduced after treatment using either the highest dose of PCB118 or the two studied doses of PCB153. The expression of *Lipin-1a* was down-regulated in a dose-dependent manner, while that of *Lipin-1b* was only barely modified by PCB118, but enhanced with only the lowest dose of PCB153. ATGL expression was also barely modified by PCBs and that of *FABP-4* was slightly reduced in the presence of five micromolars of PCB118, but it was induced by PCB153. The expression of the *FAS* gene was also impaired by PCB118 and PCB153, that of *HSL* was either not modified or only slighlty reduced, while that of *SREBP* was impaired by the lowest dose of PCB118 and the two studied doses of PCB153.

Both, PCB118 and PCB153, enhanced the BaP-induced down-regulation of the expression of *PPARγ*, *Glut-4*, *Lipin-1a* and *Lipin-1b* genes, while the two compounds caused opposite effects on *Glut-1* expression. In the same way, PCB118 and PCB153 enhanced the BaP-induced decrease of *ATGL*, *FABP4*, *FAS*, and *HSL* expression but not that of *SREBP*.

**Figure 5:**
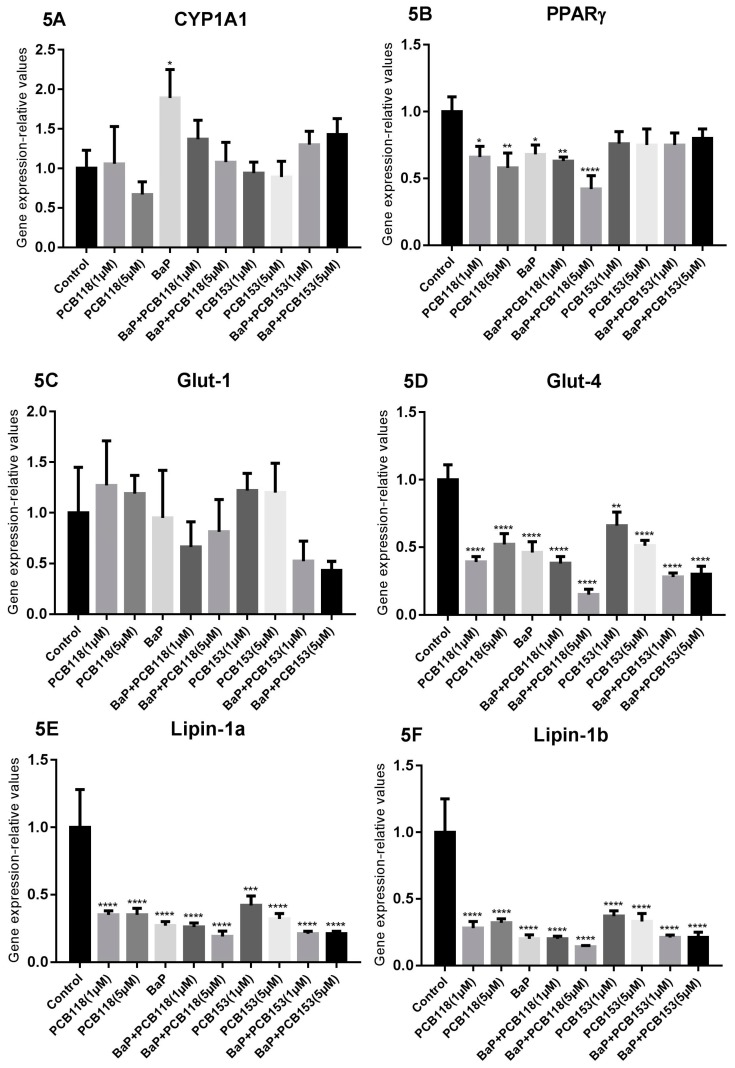

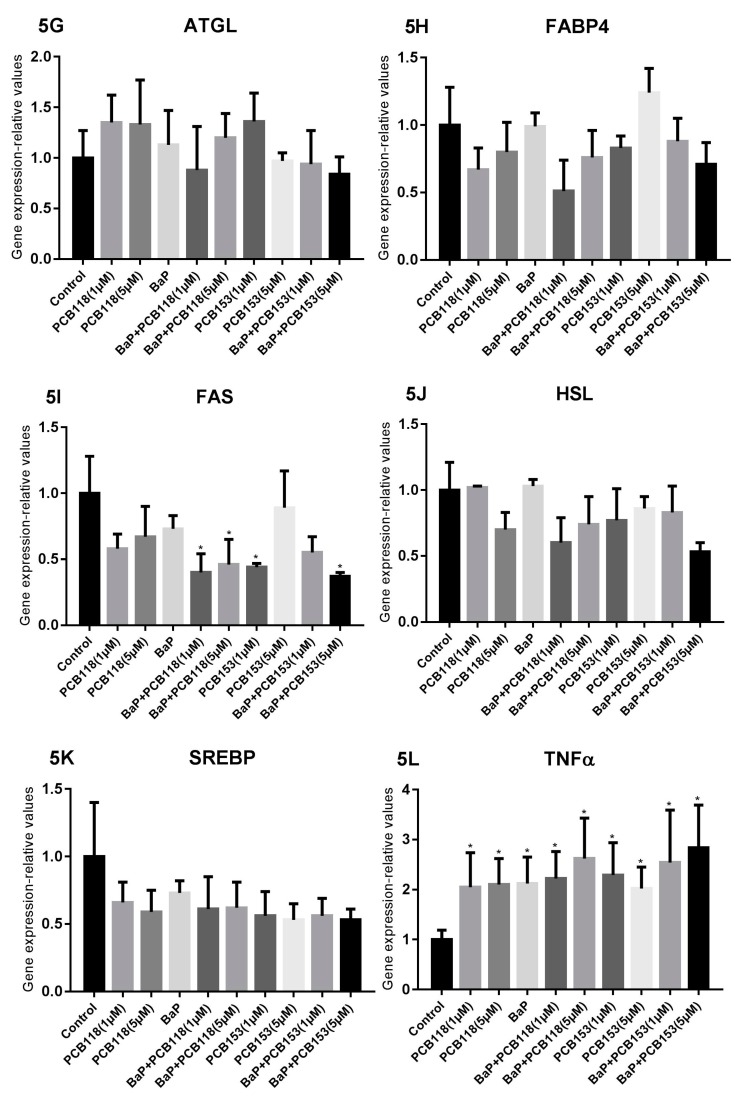

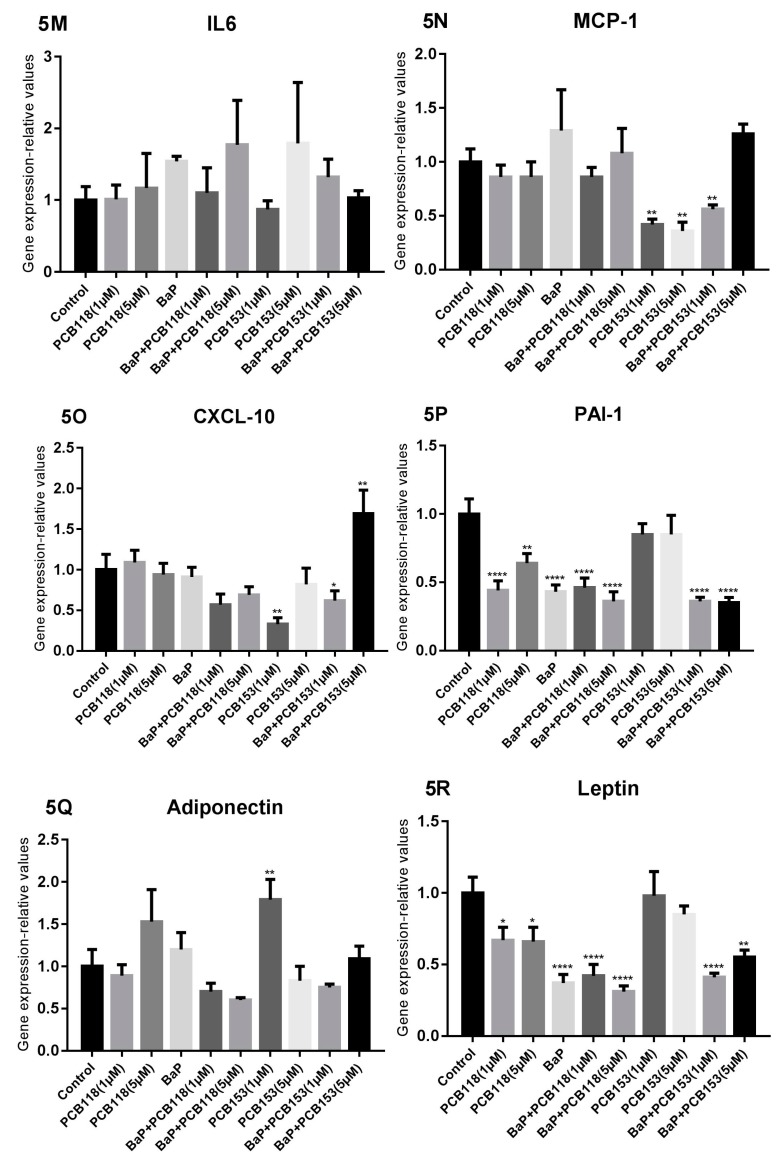
Effects of PCB118, PCB153 and BaP in “Time 2” conditions on the expression of genes related to adipocyte differentiation, glucose and lipid homeostasis and inflammation. 3T3-L1 cells were incubated with or without PCB118 (one or five micromolars) or PCB153 (one or five micromolars) in the presence or not of BaP (one micromolar) according to the “Time 2” conditions as described in materials and methods. Cells were washed twice with PBS and mRNA extraction and qRT-PCR were performed as described under materials and methods. Each incubation condition was carried out in quadriplicate and the experiment was repeated three times with similar results. The data are mean ± SD. The level of significance is given by comparison of treated trials with controls (*n* = 4 per group): * *p* < 0.05; ** *p* < 0.01; *** *p* < 0.001; **** *p* < 0.0001.

By adding the different compounds during the adipocyte differentiation, i.e., “Time 2” treatment, we can see (Figure 5) that *PPARγ* expression was slightly reduced by BaP and PCB118 alone or by the mixture of the two compounds. Compared to “Time 1”, in the “Time 2” treatment, the effects of PCB153 tested alone or mixed with BaP were slightly lower. Similar, but more pronounced effects were observed concerning the *Glut-4* expression, for which treatment with BaP enhanced PCB118 and PCB153-induced effects. *Glut-1* expression was poorly modified in response to treatment with either BaP, PCB118, PCB118 + BaP, or PCB153, while a dose-dependent reduction of the expression was observed by co-exposure with PCB153 and BaP. The expression of *Lipin-1a* and *Lipin-1b* was dramatically reduced following the treatment with either compounds: BaP, PCB118, or PCB153. Results obtained in response to a co-exposition were similar to those induced by BaP alone. The expression of *ATGL* and *FABP4* was not significantly modified by any of the treatments. *FAS* expression was poorly modulated by BaP alone but was reduced after exposure to low doses of PCB118 or PCB153. The BaP-induced decrease of *FAS* expression was slightly amplified in the case of a co-exposure with either PCB118 or PCB153.

### 2.5. Effects of PCB118, PCB153 and BaP on Gene Expression in Undifferentiated 3T3-L1 Cells

**Figure A2:**
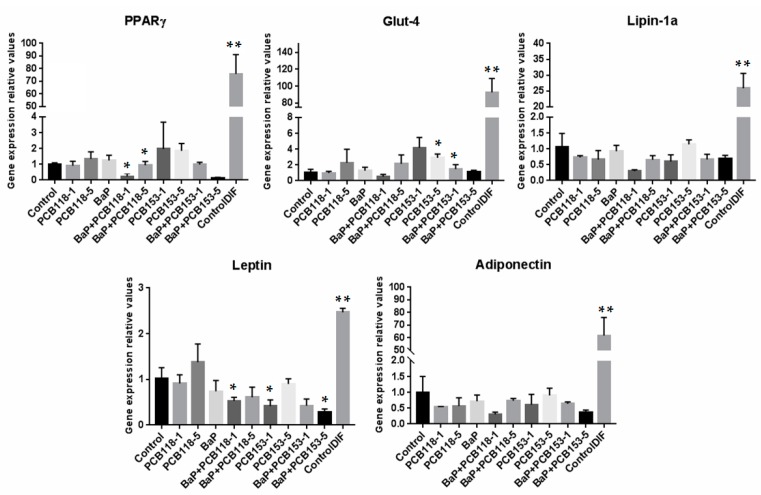
Effects of PCB118, PCB153 and BaP on undifferentiated 3T3-L1 cells on the expression of various adipogenesis related genes. Undifferentiated 3T3-L1 cells were incubated with or without PCB118 (one or five micromolars) or PCB153 (one or five micromolars) in the presence or not of BaP (one micromolar). Cells were washed twice with PBS and mRNA extraction and qRT-PCR were performed as described under materials and methods. Each incubation condition was carried out in quadriplicate and the experiment was repeated three times with similar results. The data are mean ± SD. The level of significance is given by comparison of treated trials with controls (*n* = 4 per group): * *p* < 0.05; ** *p* < 0.01.

As anticipated, the basal level of expression of genes related to adipogenesis, appeared strongly lower in undifferentiated 3T3-L1 cells than in cells that were differentiated or being differentiated: (around ×1/90 for *Glut-4*, 1/60 for *Adiponectin*, 1/25 for *Lipin-1a* and 1/70 for *PPARγ*). The level of Leptin appeared lesser reduced (around 1/2.5) (see Appendix A, Figure A2).

### 2.6. Effects of PCB118, PCB153 and BaP on Expression of Inflammatory Genes

It is now well established now that adipose tissue expresses many pro- and anti-inflammatory cytokines. The existence of a link between pro-inflammatory cytokines produced by adipose tissue and insulin resistance factors has been already highlighted [15,16,17,18,19].

**Figure 6:**
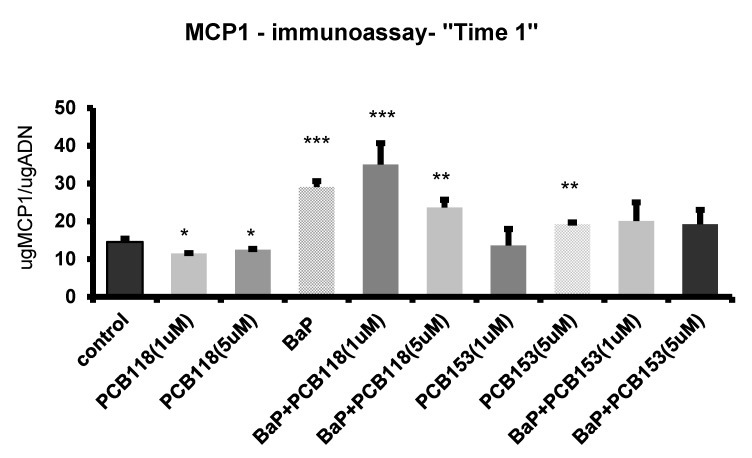
MCP1 immunoassay “Time 1”. 3T3-L1 cells were incubated with or without PCB118 (one or five micromolars) or PCB153 (one or five micromolars) in the presence or not of BaP (one micromolar) according the “Time 1” conditions described in materials and methods. The conditioned medium was collected and MCP1 peptide was measured by immuno-assay as reported under materials and methods. Each incubation condition was carried out in triplicate and the experiment was repeated three times. The values are mean ± SD. The level of significance is given by comparison of treated trials with controls (*n* = 4 per group): * *p* < 0.05; ** *p* < 0.001; *** *p* < 0.0001.

Addition of the studied pollutants at the early stage of the induction process (“Time 1” treatment) prompted an up-regulation of the expression of inflammatory genes (*TNFα*, *IL6*) (Figure 4). A synergistic effect was observed when PCB153 (but not PCB118) and BaP were added together to the cell culture. Concerning *MCP-1* gene (Figure 4), the large enhancement of transcripts, induced by BaP, was partially antagonized by PCB153 but not by PCB118. Similar results were obtained at the protein level (Figure 6), except that, concerning the high dose of PCB153 which only slightly induced the MCP-1 protein secretion. In contrast, the expression of *CXCL-10* (Figure 4) was only slightly reduced in the presence of either BaP or PCB118, but a co-exposure to a mixture of BaP and one or the other PCB (PCB118 or PCB153) induced a synergistic decrease of the expression. Surprisingly, whatever the kind of treatment used in “Time 1”, either a low down-regulation or no modification of *PAI-1* expression (Figure 4) was observed.

The expression of inflammatory genes (*TNFα*, *IL6*, *CXCL-10*, and *MCP-1*) appeared also up-regulated in response to BaP added in “Time 2” treatment, while that of PAI-1 was repressed (Figure 5). In this condition of treatment, exposure of cells to PCB118 led also to an enhancement of *TNFα* gene expression and a decrease of that of *PAI-1*, while the exposure of the cells to PCB153 caused a down-regulation of the expression of both *CXCL-10* and *MCP-1*, but induced no change in the expression of *PAI-1*. The BaP-induced up-regulation of these genes was hindered by PCB153, while the BaP-induced down-regulation of *PAI-1* was not prevented.

### 2.7. Effects of PCB118, PCB153 and BaP on the Expression of the Main Genes of Adipokines

Adipokines are signaling molecules secreted by the adipose tissue. Leptin and adiponectin are the two most abundant adipokines. Leptin is generally referred to as “the hormone of satiety” that helps to regulate the energy balance by inhibiting hunger [20]. Adiponectin is the most abundant peptide secreted by adipocytes. The reduction of this secretion plays a central role in obesity-related diseases, including insulin resistance/T2D and cardiovascular diseases [21].

“Time 1” treatment (Figure 4) by either BaP alone or a mixture of BaP and PCBs, in which cells were treated during the induction process, led to a very strong repression of both *leptin* and *adiponectin* expression, which was almost totally suppressed. In contrast, in the presence of the high concentration of PCB118 or PCB153 alone, only a faint down-regulation of the adipokine genes expression was detected.

However, regarding *adiponectin* and *leptin* expression, the effects observed in “Time 1” were not detected in “Time 2” treatment in which BaP induced only a modest up-regulation of *adiponectin* expression (Figure 5), whereas the *leptin* expression was down-regulated by exposure to either BaP, BaP + PCB118, or BaP + PCB153, but was poorly modified by exposure to PCB118 or PCB153 alone.

### 2.8. Summary of the Cocktails Effets of PCB118, PCB153 and BaP and Effects on AhR Transcriptional Activity

The Table 1 sums up qualitatively the synergic or antagonist effects of BaP versus PCB + BaP and of PCB versus PCB + BaP. The significance of all different synergic and antagonist cocktail effects of PCB and BaP which are reported in Table 1 and Figure 4 and Figure 5 is presented in Table A1, Appendix A. Generally, the effects obtained following cell exposure to the studied pollutants, BaP and PCBs, were more pronounced when they are tested in “Time 1” condition. When cells were co-exposed to BaP and PCBs, most genes belonging to the adipogenesis process (*PPARγ*, *Lipin-1a*, *Lipin-1b*, *SREBP FAS*, and *HSL*) were synergistically down-regulated in both cell treatment periods. Similarly, PCBs potentiated the BaP-induced down-regulation of *Glut-4*. Inversely, inflammatory genes (*TNFα*, *IL6*, *MCP-1*, *adiponectin*, and *leptin*) were synergistically up-regulated at “Time 1”. Other inflammatory genes such as *MCP-1*, *CXCL-10* and *IL6* at “Time 2” or *Glut-1* at “Time 1” were antagonistically regulated by BaP and PCB153.

### 2.9. Effects of PCB118, PCB153 and BaP on Xenobiotic Responsive Element (XRE)-Controlled Luciferase Activity

**Figure 7:**
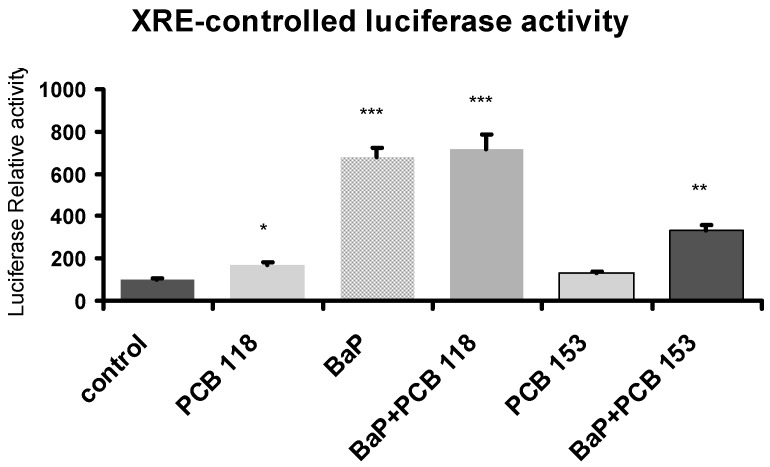
Effects of PCB118, PCB153 and BaP on AhR transcriptional activity. Following transfection with a luciferase reporter gene controlled by a xenobiotic esponsive element (XRE) sequence, cells were challenged with PCB118 (five micromolars) or PCB153 (five micromolars) in the presence or not of BaP (one micromolar) according to “Time 1” conditions reported under materials and methods. The values are represented as mean ± SD. *n* = 4 per group. The level of significance is given by comparison of treated trials with controls (*n* = 4 per group): * *p* < 0.05, ** *p* < 0.01, *** *p* < 0.001.

PCB118—a dioxin-like PCB—and BaP are two potent AhR agonists [5], while PCB153—a bulky PCB—activates the PXR/CAR factors [6]. In order to get some insight on the involvement of AhR pathway in the mechanism of action of these compounds, cells were transiently transfected with the reporter plasmid XRE-Luc, in which luciferase expression is driven by AhR, and exposed at “Time 1”. Results are summarized in Figure 7. As expected, as compared to control, both compounds PCB118 and BaP significantly induced luciferase expression. On the other hand, PCB153 caused by itself a weak effect, but when added mixed with BaP, this PCB counteracted the BaP-induced luciferase expression.

## 3. Discussion

Epidemiological studies have allowed the association of environmental exposure to PCB (mainly through food, notably fishes) to an increased risk of T2D development [3,4]. Recently, we have studied the effect of PCBs in mice exposed for one month to PCB118 and/or PCB153 and we have shown that adipose tissue is the main target organ for PCBs-induced metabolic detrimental effects. In this work, using transcriptomic analyses, we have highlighted the down-regulation of two genes involved in insulin sensitivity and glucose homeostasis: *Glut-4* and *Lipin-1*. However, we did not observe any induction of inflammation target genes, such as pro-inflammatory cytokines and chemokines [9].

Recent studies support a role of adipose tissue and inflammation in the regulation of insulin signaling pathways. Hotamisligil and colleagues have first demonstrated that inflammatory mediators are involved in insulin signaling pathways [15]. Adipose tissue acts as a site for the production of inflammatory mediators and adipokines such as IL6, IL1β, TNFα, leptin, resistin, MCP-1, PAI-1, visfatin and adiponectin [15,16,17,18,19].

In order to clarify the health consequences of environmental and food co-exposure to PAHs that exert mutagenic and pro-inflammatory effects [10,11,12]. We have studied the cocktail effects of BaP and PCBs—PCB118 or PCB153, which are a PCB-DL and a bulky PCB respectively—on adipose physiology, using the 3T3-L1 cell line.

Firstly, we studied the effect of these organic pollutants on the adipose differentiation process, by measuring the cytosolic lipid content and evaluating *Lipin-1a*, *Lipin-1b*, and *PPARγ* expression. PPARγ is indeed considered the “master regulator” of adipogenesis [14] and recent data suggest that Lipin-1 functions as a key regulator of PPARγ activity, through its ability to release co-repressors and recruit co-activators [22]. The greatest effects were observed, when treatments were carried out during the differentiation process (“Time 1” treatment), while the effects were weaker when the exposure was performed on differentiated adipocytes (“Time 2” treatment). Similar results had been obtained with PCB126 (a PCB-DL) by Gadupudi and coworkers [23], who demonstrated that pre-exposure of preadipocytes to PCB126 resulted in a significant, dose-dependent reduction in their differentiation state, and this effect was larger than that observed in cultures treated with PCB126 during the differentiation process. Our data show that both BaP and PCB118 reduce cytoplasmic lipid content; *Lipin-1a*, *Lipin-1b* and *PPARγ* expression, while PCB153, at the two studied doses, down-regulates the lipid content and *Lipin-1a* expression while it enhances *PPARγ* and *Lipin-1b* expression. These data are in accordance with previous in vitro studies performed on adipocytes using PCBs-DL (PCB126) or bulky PCBs (PCB153) [23,24]. Interestingly, PCB118 and PCB153, strengthened the BaP induced-effect on the decrease of *Lipin-1a*, *Lipin-1b*, and *PPARγ* expression, while they repressed its effect on lipid content. This repression of *Lipin-1a*, and *Lipin-1b* expression induced by BaP and/or PCBs could result from the induction of pro-inflammatory cytokines and by the enhancement of endoplasmic reticulum stress. Lipin-1 is indeed a multifunctional protein which plays a critical role in adipose differentiation, mitochondrial oxidation, and triglyceride synthesis. Down-regulation of this factor was tightly associated to the insulin resistance in mice and human [25,26]. Although some cytokines such as TNFα and IL1β reduce adipose *Lipin-1* expression [27], the mechanism by which the expression of *Lipin-1* was impaired in adipose tissue in obesity states remains unclear. Recently, endoplasmic reticulum stress has been shown to be involved in the pathogenesis of obesity through suppression of the Lipin-1 expression in 3T3-L1 adipocytes [28]. On the other hand, cytochromes P450, notably those belonging to the CYP1 family, can induce endoplasmic reticulum stress [29], and interestingly, our results show that combination of BaP and PCB118 induces *CYP1A1* expression in adipocytes.

Our results show also that the decrease of lipid content and *PPARγ* expression is associated with a decrease of other genes involved in lipid homeostasis, such as *FAS*, *ATGL*, *HSL*, *SREBP*, and *FABP-4*. The effects of BaP were larger than those induced by PCBs alone, which induced similar but lower effects on gene expression, except for *FABP-4*, for which expression was enhanced by PCB153. Liver X receptors (LXRs) are nuclear receptors that control cellular metabolism and modulate the expression of genes involved in cholesterol and lipid metabolism. Activation of LXR in adipocytes leads to the induction of the expression of *SREBP*, *FAS*, and *HSL* [30]. PAHs down-regulate *FAS* and *SREBP* expression in HepG2 cells throughout the inhibition, via AhR, of LXR transactivation [31]. Such a process would probably occur also in adipocytes and so it could explain the observed down-regulation of *FAS*, *HSL*, *SREBP* induced by BaP and PCB118.

The induction of FABP-4 by PCB153 observed in our study is probably related to the induction of PPARγ. Indeed, it has been demonstrated that PPARγ binds to A (adipocyte-type)-FABP-PPRE [32] and up-regulates its expression.

Most mammalian cells import glucose by a process of facilitated diffusion mediated by members of the Glut family of membrane transport proteins that play a major role in glucose homeostasis. Therefore we have studied the effect of BaP and/or PCBs on *Glut-1* and *Glut-4* expression. Our data demonstrated that, in pre-adipocytes, BaP represses *Glut-4* expression and induces that of *Glut-1*. The BaP-induced decrease of *Glut-4* expression was enhanced by either PCB118 or PCB153, while the BaP-induced *Glut-1* expression was enhanced by PCB118 only. In mature adipocytes, we observed that BaP and also PCB118 or PCB153 (alone or in combination with the former) decreased *Glut-4* expression. It is established that Glut-4 mediates insulin-stimulated glucose uptake in adipocytes. Under insulin resistant states, such as obesity and T2D, *Glut-4* expression is down-regulated specifically in adipose tissue but not in skeletal muscle [33]. Glut-1 catalyzes the rate-limiting step in supplying cells of the central nervous system with glucose, an essential fuel for these cells. Glut-1 is frequently up-regulated during oncogenesis in many different tissue types, a process that is probably essential for tumors to grow beyond a size limited by their glycolytic capacity (the Warburg effect) [34]. In brown adipose tissue, Glut-1 is involved in the norepinephrine-induced glucose uptake [35]. In our data, the main effects of PAHs and PCBs in adipose tissue on the Glut transporter family was on the Glut-4 isoform. This observation is in agreement with the usually observed association of the down-regulation of this gene with insulin resistant states.

T2D and insulin resistance are linked to a chronic low-grade inflammation of adipose tissue [15,16,17,18,19]. Our data show that in pre-adipocytes (“Time 1” treatment), BaP enhanced the expression of pro-inflammatory cytokines (*TNFα*, *IL6*) which was associated with an enhanced production of the chemokine MCP-1. The effect of PCBs on *MCP-1* expression was low, but interestingly the lowest dose (1 µM) was the more potent. While PCB118 did not modulate the pro-inflammatory effect of BaP, PCB153 enhanced the BaP-induced TNFα and IL6 production. These data are in agreement with the literature. Studies based indeed on differentiated human THP1 macrophages have led to propose that after the binding to some ligands, such as TCDD, AhR activates the EGFR-pathway, leading to ERK1/2 activation, through which expression of the *TNFα* gene is up-regulated [36]. In addition, toxic metabolites derived from AhR ligands, such as BaP, may also activate a range of signaling pathways, including ERK1/2 [37]. The regulation of *IL6* expression in response to AhR ligands appears to be rather complex and is depending on the cell type and the cytokine/inflammatory signal environment. In MCF-7 and CV-1 cells, it has been demonstrated that AhR plays a role on *IL6* expression through the de-repression of the *IL6* promoter, leading to a synergistic *IL6* expression in the presence of other inflammatory signals, such as IL1β [38]. Induction of *IL1β* and *IL6* expression in adipose tissue has been recently described, after mice exposure to PCB153 [39]. It is likely that the de-repression of the *IL6* promoter is involved in the BaP- and PCB-induced up-regulation of *IL6* expression. The induction of pro-inflammatory cytokines we have observed, is also associated to an increase of *MCP-1* expression. Similar results have been described in mice exposed to the PCB-DL, PCB77 [40]. Moreover, MCP-1 is a key chemokine expressed by the adipocytes and plasma level of this factor positively correlates with the increased adiposity. The secretion of MCP-1 is sufficient to induce the recruitment and infiltration of macrophages in the adipose tissue which lead to the initiation of the inflammatory response and the obesity-related insulin resistance [41].

Pro-coagulant and fibrinolytic markers have been proposed as being risk factors for the development of T2D. When fed with a high-fat/high carbohydrates diet, mice homozygous null for the *PAI-1* gene display ameliorated insulin and glycemic measures and protective effects against the development of obesity and insulin resistance as compared with wild-type mice. Likewise, early cross-sectional studies in humans have reported correlations between elevated PAI-1 concentrations and obesity, insulin resistance, impaired glucose tolerance, and T2D [42]. Our data show that BaP and PCBs induced no significant modification of *PAI-1* expression in pre-adipocytes (“Time 1” treatment). Inversely, in mature adipocytes (“Time 2” treatment), BaP and PCB118 but not PCB153 down-regulated *PAI-1*. This latter data is consistent with a previous study performed in vivo in mice, in which it was demonstrated that PCB153 slightly enhances PAI-1 blood circulating level [43].

Adipose tissue secretes also specific cytokines, called adipokines, such as the two most abundant: leptin and adiponectin. Our results show that in pre-adipocytes (“Time 1” treatment), BaP markedly represses *leptin* and *adiponectin* expression. PCBs alone reduce only barely their expression, and enhance the effect of BaP. Comparable but less pronounced effects were observed when treatment was performed on mature adipocytes (“Time 2” treatment). Similarly, it has been demonstrated in 3T3-L1 pre-adipocytes, that PCB77 (a PCB-DL) and to a lesser extent PCB153, reduce *adiponectin* expression [44]. Moreover, recently, it was observed in pig ovarian cells co-cultures, a BaP-induced decrease of adiponectin secretion and receptors expression [45]. Adiponectin, secreted by the adipocytes, is considered as an insulin-sensitizing, anti-apoptotic, and anti-inflammatory peptide. Adiponectin secretion seems to be regulated by local insulin sensitivity [46]. Leptin is involved in the regulation of food intake through the central nervous system. This adipokine exerts both pro-inflammatory activity and insulin-sensitizing actions [46]. Chronic leptin replacement therapy has been shown to reverse liver and muscle insulin resistance and fasting hyperglycaemia in patients displaying a severe lipodystrophy [47]. Taken together, these data suggested that by inducing a decrease of *adiponectin* and *leptin* expression, BaP and PCBs are also potent to promote the development of insulin resistance states.

**Figure A3:**
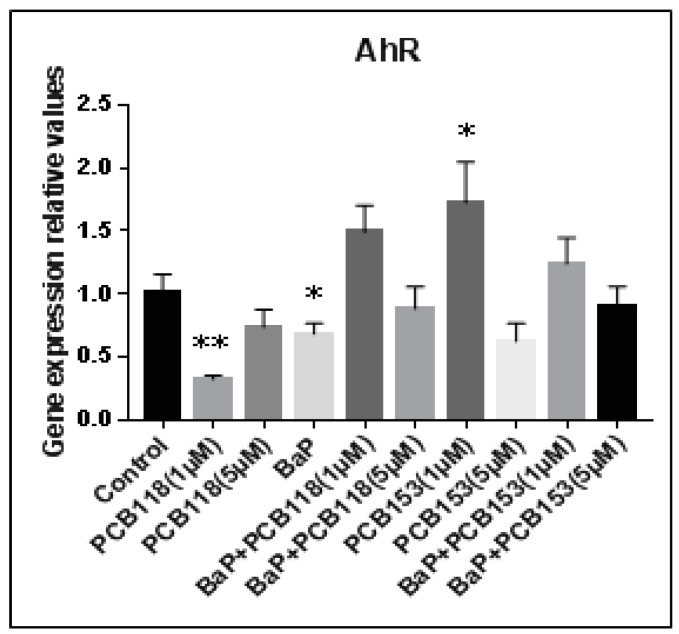
Effects of PCB118, PCB153 and BaP in “Time 1” conditions on the expression of *AhR*. 3T3-L1 cells were incubated with or without PCB118 (one or five micromolars) or PCB153 (one or five micromolars) in the presence or not of BaP (one micromolar) according to the “Time 1” conditions described in materials and methods. Cells were washed twice with PBS and mRNA extraction and qRT-PCR were performed as described under materials and methods. Each incubation condition was carried out in quadriplicate and the experiment was repeated three times with similar results. The data are mean ± SD. The level of significance is given by comparison of treated trials with controls (*n* = 4 per group): * *p* < 0.05; ** *p* < 0.01.

The permanent environmental exposition to a range of chemical compounds to which we are subject makes it advisable to evaluate possible resulting cocktail effects on cell function and health. As mentioned in Table 1, most genes belonging to adipogenesis (*PPARγ*, *Lipin-1a*, *Lipin-1b*, *SREBP FAS*, and *HSL*) were synergistically down-regulated at “Time 1” or “Time 2” treatment. Similarly, PCBs potentiated BaP-induced down-regulation of *Glut-4*. Inversely, inflammatory genes (*TNFα*, *IL6*, *MCP-1*, *adiponectin*, and *leptin*) were synergistically up-regulated at “Time 1”. Other inflammatory genes as *MCP-1*, *CXCL-10* and *IL6* at “Time 2” or *Glut-1* at “Time 1” were antagonistically regulated by BaP and PCB153. From a molecular point of view, BaP and PCB118 are potent agonists of AhR [5] and PCB153 activates two other transcription factors PXR and CAR [6]. In order to get some insight on the involvement of AhR pathway, cells were transiently transfected with the reporter plasmid XRE-Luc, in which luciferase expression is driven by AhR, and exposed to the “Time 1” treatment. BaP largely enhanced luciferase expression, and PCB118 or PCB153 alone induced only a slight increase. We have observed that PCB153 induces *AhR* transcripts, while BaP and PCB118 reduce it (see Figure A3 in Appendix A). When BaP is used in conjunction with PCB118 we observed an additive effect, inversely PCB153 antagonized in part BaP effects. CAR and PXR belong to the family of steroid nuclear receptors and in the absence of ligand, these receptors repress the transcription of target genes via recruitment of the corepressors, SMRT and N-CoR. In turn, these corepressors recruit various histone deacetylases resulting in an inactive chromatin state. Hormone binding triggers the release of corepressors and the subsequent association of an array of coactivators, including SRC-1, CBP/p300 and ASC-2 [48]. Previously, we have demonstrated that retinoids decrease the CYP1A1 induction [49]. This process is related to the AhR pathway through the release of the corepressor SMRT by RAR, and its subsequent dimerization with AhR. Probably, such a process is involved in the observed antagonism effect of PCB153 on BaP-induced luciferase expression. Moreover, it was recently described that PXR protects from BaP-induced DNA damage, and it can bound AhR at its DNA-binding domain, and this association may play a role in preventing of the AhR from binding to its target genes [50]. Inversely, concerning the expression of *CYP1A1*, *TNFα*, and *IL6*, we observed that PCB153 enhances the effect of BaP in pre-adipocytes (“Time 1” treatment). Interestingly, it has been described that AhR regulates CAR expression in both murine and human liver, and such a phenomenon could also occur in adipose tissue [51]. In the same way, PAH such BaP, and their dihydrodiol metabolites, were able to activate PXR and enhance *CYP3A4* expression in human cell lines (HepG2 and HEK-293 cells) [52]. Therefore, the enhancement of BaP-induced effects by PCB153 could be interpreted at least in part on the one hand by the activation of PXR by BaP and their dihydrodiol metabolites and on the other hand by the enhancement of *CAR* expression through the AhR pathway.

Generally, the effects obtained after exposure to BaP and PCBs were more pronounced in pre-adipocytes (“Time 1” treatment) than in mature one (“Time 2” treatment). These data are in agreement with the lower level of AhR protein already observed during the ongoing adipose differentiation in 3T3-L1 cells [53]. As discussed above, AhR activation enhances in adipocytes the expression of both pro-inflammatory cytokines (TNFα, IL6) and MCP-1 chemokine, which can in return amplify the inflammatory signal by inducing the recruitment and infiltration of macrophages in this tissue. However, according to recent studies, the AhR is essential for the differentiation and activation of T helper 17 (Th17) cells. PAHs, such as BaP, halogenated-dioxins and their congeners, such as PCB-DL, contribute to the inflammation via the promotion of the Th17 differentiation [54]. Interestingly, *SREBP*, which is an LXR targeted-type gene, mediates also the suppression of Th17 differentiation by binding to the E-box element found on the *IL17* promoter which lead to a physical interaction with AhR and an inhibition of the AhR-controlled *IL17* transcription [55]. Th17 enrichment has been observed in both adipose tissue of obese subjects and, obese type 2 diabetic patients and also in adipose tissue-derived stem cells from obese subjects. Such an enhancement of Th17 cells contribute to adipose tissue inflammation and lead to reduce the insulin response by promoting Th17 and monocyte activation [56]. Therefore the down-regulation of *SREBP* by BaP and/or PCBs would promote Th17 differentiation and may reshape the regulation of the inflammation process.

Taken together, these data suggest that PAHs and PCBs-DL can promote T2D through the modulation of the balance between two kinds of cellular process: on the one hand, inhibition of adipocyte differentiation and on the other hand, the development of a low grade chronic inflammation of adipose tissue. This process occurs through targeting the AhR pathway both in adipose tissue and immune system. We demonstrated that in mice-derived adipose cells, PAHs modulate more deeply than PCBs the down-regulation of ADGG and the induction of INFG. However, when studying the cocktail effects, our data suggest that PCB-DL, such as PCB118, enhance in adipose tissue the BaP-induced effects while, depending on the gene studied, bulky PCB, such as PCB153, increase or repress them. Therefore, it appears really essential to study the cocktail effects. Studies using a single pollutant are indeed poorly representative of the environmental context and could conduct to false leads. Moreover, in vivo, in the context of the entire body, the organs interact with one another. For example, thyroid, which regulates metabolic processes essential for normal growth and development as well as the metabolism steady state in the adult, plays an essential role in adipose tissue function. Dioxin-like compounds that are potent AhR agonists, has been demonstrated to affect thyroid endocrine functions by causing hypo-thyroxemia associated to a reduced blood level of thyroxin (T4). This reduction is at least in part the consequence of some dysfunctions, such as an increase of thyroxin glucuronidation in liver, a down-regulation in the expression of sodium iodide symporter (*NIS*) and *cathepsin B* in thyrocytes. All this leads to the development of thyroid autoimmune disorders by modulating immune response through AhR pathway [57,58]. Therefore, it would be of interest to perform in vivo co-exposition to PAH and PCB in mice to evaluate the consequence in the main target organs—adipose tissue, liver—and on thyroid function.

## 4. Materials and Methods

### 4.1. Chemicals and Reagents

Foetal calf serum (FCS) (‘HyClone’, lot n°CPB 0054) was from Perbio (Thermo scientific, Brebières, France). The Dulbecco’s Modified Eagle Medium (DMEM) and newborn bovine serum (NBBS) lot n° S 857 46S0750 were from Dutscher (Brumath, France). The lipofectin reagent and the luciferase detection kit was from Promega (Charbonnières, France). The following compounds were from Sigma (L’Isle d’Abeau, France): Benzo-apyrene (BaP), DMSO, phosphate-buffered saline (PBS), bovine serum albumin (BSA), isobutyl-methylxanthine (IBMX), Insulin (bovine origin), Dexamethasone (DEX), 3-(4,5-dimethylthiazol-2-yl)-2,5-diphenyl tetrazolium. (MTT) and Oil Red O reagent (ORO). Proteinase-K was from Invitrogen (Cergy_Pontoise, France), BioRad kit for protein assays was from Interchim (Montluçon, France), M-MLV Reverse Transcriptase from In Vitrogen/Thermo-Fischer (Cergy-Pontoise, France), 5× HOT Pol Evagreen^®^ qPCR Mix Plus was from Euromedex, (Souffelweyersheim, France) PCB118 and PCB153 were purchased from AccuStandard Inc. (New Haven, CT, USA).

### 4.2. Cell Culture and Differentiation

The preadipocyte of cloned cell line 3T3-L1, isolated from Swiss strain of mouse embryos, was obtained from American Type Culture Collection (ATCC). The 3T3-L1 were routinely cultured at 37 °C, in a humid atmosphere of 5% CO_2_ under sterile condition in 25 or 75 cm² flask filled with a basic cell culture medium containing DMEM supplemented with 10% NBBS and 1% penicillin/streptomycin solution at 5 mg/mL. The medium was changed every four days and the cells are reseeded imperatively before reaching 50–60% confluence.

Cell differentiation was carried out following seeding of cells in multiwell plates: 24-wells plate was seeded with 25,000 cells/well or 100,000/well in 6-wells plate. When cells reached 70–80% confluence, that day is called day 0 and the medium was changed by the FCS-basic medium containing FCS instead of NBBS. At day 2, the medium was changed by the induction medium (FCS-basic medium supplemented by 0.5 mM IBMX, 1 μM DEX and 1 μg/mL insulin). At day 4, the medium was replaced by the differentiation medium (FCS-basic medium containing 1 µg/mL insulin), and this medium are changed in every three days until day 14 (Figure 1).

### 4.3. Cell Treatment

In order to test the compounds BaP, PCB118 and PCB153, alone or mixed, these were dissolved in DMSO at different concentrations. These working solutions were added to the culture medium in order to reach final concentrations of 1 and 5 µM for PCB118 and PCB153, and 1 µM for BaP, respectively. The solvent (DMSO) was added in assays in order to reach everywhere the same concentration of solvent (0.5 µL/mL) in each assay.

According to experiments, PCBs and BaP were incorporated in the culture medium at two different times: either at the onset of induction period “Time 1” (day 2 to day14) in order to highlight a possible impact of PCBs on adipocyte differentiation and maturation or “Time 2” (day 4 to day 14) in order to evidence an effect of PCBs on only the maturation of adipocytes.

### 4.4. MTT Test

The MTT test was used to evaluate a possible effect of PCBs and BaP on cell survival and avoid cytotoxic doses of these compounds.

After cells incubation with MTT for about 3 h at 37 °C, the cells, their mitochondria and the violet precipitates formazan are dissolved in 100% DMSO. The optical density (OD) at 550 nm led to know the relative amount of alive and active metabolically cells.

### 4.5. Oil Red O Staining (ORO) and Quantification of Lipids

This method allows to observe the evolution of adipocyte differentiation identified by the hydrophobic lipid staining in liquid phase. The colored body, called lysochrome, moves from the solvent where it is located into lipid tissue. The commercial ORO solution is diluted the day before and extemporaneously filtered according to standard methods [59].

For staining, after removing the culture medium, culture dishes (24 wells plates) were washed with warm PBS and the cells were fixed with Para-Formaldehyde (4% in PBS) for 30 min and finally three washes with PBS was carried out and the cells were dried. Then 300 μL of diluted ORO solution was added in. After incubation for 2 h at 37 °C and three times washing with water, cell staining was extracted in isopropanol (300 µL). The quantification was done by measuring the OD at 492 nm.

In order to express the results in relation to the cellular material, the measured OD is normalized to the amount of DNA. For this, the cells were lysed using a buffer (100 µL) containing 0.01 M Tris pH 7.8, 0.2 M NaCl, 0.005 M EDTA, 0.5% Sodium dodecyl sulfate (SDS), supplemented with 20 mg/mL proteinase-K. DNA was quantified by spectrophotometry using the Nanodrop^®^ assay. (100 µL) containing 0.1 M Tris pH 8.0, 0.2 M NaCl, 0.005 M EDTA, 0.5% Sodium dodecyl sulfate (SDS), supplemented with 20 mg/mL proteinase-K. DNA was quantified by spectrophotometry using the Nanodrop^®^ assay.

### 4.6. Immuno-Detection of MCP-1

MCP-1 detection was carried out on the cell culture medium using the ‘Mouse MCP-1 Elisa Kit’ from Thermo-scientific and according to the instructions of the manufacturer. For assays, cell medium was usually 4 times diluted and measures were carried out on 100 µL of such dilutions. MCP-1 concentration was found between 4 and 20 ng/mL of culture medium.

### 4.7. RNA Isolation and qRT-PCR Analyses

Total RNA was extracted from 3T3-L1 cells using Trizol^®^ as the solvent of lysis and quantified by spectrophotometry. The conversion of RNA to cDNA is carried out in the presence of nucleotides (dNTPs), random primers, reverse transcriptase (M-MLV Reverse Transcriptase from Invitrogen, Paris, France) and incubation buffer. The cDNA was used as a template for PCR to amplify the specific product.

PCR experiments were performed using a LightCycler480 System (Roche, Boulogne-Billancourt, France) in 96 well-plates. The PCR was performed with 0.4 μM of each primer and 5× HOT Pol Evagreen^®^ qPCR Mix Plus (Euromedex, Souffelweyersheim, France). Cycling conditions including initial PCR activation (1 cycle) at 95 °C for 15 min and followed by 40 cycles of amplification (15 s denaturation at 95 °C, 20 s primer annealing and 20 s fragment elongation at 72 °C). A melt program was performed at the end of the PCR. The raw fluorescence data were analyzed using LightCycler480 (Roche Diagnostics, Meylan, France). The sequences of primers used for c-DNA amplification by qPCR are listed in Table 2. Target gene *mRNA* expression was normalized to β-actin, and data were quantified using the 2^−ΔΔ*C*t^ method [60].

### 4.8. Transient Transfection and Luciferase Assay

Transient gene expression experiments were performed in 24-well plates according to Vallee et al., 2004 and Njock et al., 2014 [61,62]. Each well (2 cm^2^) contained 2 × 10^5^ cells in DMEM supplemented with 10% FCS. One hour before the transfection, cells were incubated in serum-free DMEM, then the transfection was carried out as using, for each well, 0.4 mL DMEM containing 0.3 µg of plasmid DNA construction to be tested including two XRE sequences driving luciferase (LUC) gene expression (XRE-TK-LUC) that was previously described [63], 0.1 µg of control plasmid driving the β-galactosidase expression, and 2 µL of lipofectin. Sixteen hours later, the medium was replaced by 1 mL DMEM supplemented with 10% FCS and culture was resumed for twenty four hours. The cells were then challenged, with or without BaP and PCB, or both compounds, and incubation resumed for additional twenty four hours. Extracts were prepared using the lysis buffer of the Promega luciferase kit. Protein content of each assay was determined (BioRad assay kit, Marnes-la-Coquette, France).

Luciferase assays were performed using 30 µg proteins, which was added to the luciferase assay reagent, according to instructions of the manufacturer (Promega, Charbonnières-les-Bains, France), and measure of light emission (1 to 5 s) was carried out using a photometer (Berthold, Thoiry, France). Results were reported as arbitrary light units of luciferase normalized for transfection efficiency relative to β-galactosidase activity. At least three independent experiments were carried out four times each.

### 4.9. Statistical Analyses

Statistical analysis was performed using GraphPad Prism software (GraphPad, San Diego, CA, USA). The intergroup comparisons were obtained with Kruskal–Wallis test followed up by Dunn’s test. Values were considered statistically different at *p* < 0.05. The results are presented as mean ± SD.

## Figures and Tables

**Table 1:** Summary of synergic or antagonist cocktail effects of BaP and PCBs on genes expression in “Time 1” and “Time 2”: ↓↓: down-regulation with BaP and down-regulation with PCBs. ↑↑: up-regulation with BaP and up-regulation with PCBs. ↓↑: down-regulation with BaP and up-regulation with PCBs. ↑↓: up-regulation with BaP and down-regulation with PCB. Inflammatory genes are in bold characters. Adipokines are in italic and bold characters. The level of significance of differences between BaP-treated and PCB-treated assays is * *p* < 0.05; ** *p* < 0.01; *** *p* < 0.001. For details see the tables of significances (Table A1 in Appendix A).

Treatment and Time		Synergism of BaP and PCB		Antagonism of BaP and PCB	
		↓↓	↑↑	↓↑	↑↓
BaP and PCB118	Time 1	PPARγ ** Glut-4 *** Lipin-1a * Lipin-1b ** ATGL ** FABP-4 ** FAS ** HSL *** SREBP * CXCL-10 ** Adiponectin *** Leptin ****	CYP1A1 ** Glut-1 **		
BaP and PCB118	Time 2	PPARγ * Glut-4 ** Lipin-1a * Lipin-1b * HSL * PAI-1 **	CYP1A1 *	***Leptin*** **	***Adiponectin*** **
BaP and PCB153	Time 1	PPARγ *** Glut-4 *** Lipin-1a ** Lipin-1b *** ATGL *** FABP-4 **** FAS *** HSL ** CXCL-10 ** Adiponectin ***	CYP1A1 ** TNFα *** IL6 **		Glut-1 *** MCP-1 ***
BaP and PCB153	Time 2	Glut-1 * Glut-4 ** Lipin-1a * Lipin-1b * FAS * HSL * PAI-1 **		***Leptin*** **	**MCP-1** ** CXCL-10 ** Adiponectin **

**Table 2:** Sequences of primers used in qRT-PCR analyses. F: forward; R: reverse.

Primers	Sequence (5′-3′)	
	F	R
Mus_Actin β	GGAGGGGGTTGAGGTGTT	GTGTGCACTTTTATTGGTCTCAA
Mus_AhR	TGCACAAGGAGTGGACGA	AGGAAGCTGGTCTGGGGTAT
Mus_Cyp1a1	TCTTTTGGGAGGAAGTGGAA	TCCATACATGGAAGGCATGA
Mus_PPARγ	AAGAGCTGACCCAATGGTTG	ACCCTTGCATCCTTCACAAG
Mus_Glut-1	GGAGAAGAAGGTCACCATC	GAGTAGTAGAACACAGCATTG
Mus_Glut-4	GAGAGAGCGTCCAATGTC	CGAAGATGCTGGTTGAATAG
Mus_Lipin-1a	GGTCCCCCAGCCCCAGTCCTT	GCAGCCTGTGGCAATTCA
Mus_Lipin-1b	CAGCCTGGTAGATTGCCAGA	GCAGCCTGTGGCAATTCA
Mus_FABP-4	GATGAAATCACCGCAGACGACA	ATTGTGGTCGACTTTCCATCCC
Mus_FAS	GTGACCGCCATCTATATCG	CTGTCGTCTGTAGTCTTGAG
Mus_ATGL	ACCAGCATCCAGTTCAAC	CGAAGTCCATCTCTGTAGC
Mus_HSL	CTGAGATTGAGGTGCTGTC	GGTGAGATGGTAACTGTGAG
Mus_SREBP	CAGCACAGCAACCAGAAGC	CCTCCTCCACTGCCACAAG
Mus_TNFα	ACAAGCCTGTAGCCCACGTCGTAGC	AATGACTCCAAAGTAGACCTGCCCGG
Mus_IL6	ACGGCCTTCCCTACTTCAC	ACAGGTCTGTTGGGAGTGG
Mus_MCP-1	CATCCACGTGTTGGCTCA	GATCATCTTGCTGGTGAATGAGT
Mus_CXCL-10	GCTGCCGTCATTTTCTGC	TCTCACTGGCCCGTCATC
Mus_PAI-1	AGGATCGAGGTAAACGAGAGC	GCGGGCTGAGATGACAAA
Mus_Leptin	CAGGATCAATGACATTTCACACA	GCTGGTGAGGACCTGTTGAT
Mus_Adiponectin	TGTTCCTCTTAATCCTGCCCA	CCAACCTGCACAAGTTCCCTT

## Abbreviations

ADG	Adipogenesis
ADGG	ADG-related genes
AhR	Aryl hydrocarbon receptor
ASC-2	Anterior suture cataract 2
ATCC	American Type Culture Collection
ATGL	Adipose triglyceride lipase
BaP	Benzo(a)pyrene
BSA	Bovine serum albumin
c/EBP	CCAAT/enhancer binding protein
CAR	Constitutive androstane receptor
CBP	CREB binding protein
cDNA	Complementary DNA
CXCL-10	Chemokine (C-X-C motif) ligand 10
CYP1	Cytochrome P450, family 1
CYP1A1	Cytochrome P450 family 1 subfamily A member 1
DEX	Dexamethasone
DMEM	Dulbecco’s Modified Eagle Medium
dNTP	Deoxynucleotide
EDTA	Ethylenediaminetetraacetic acid
EGFR	Epidermal growth factor receptor
ERK	Extracellular regulated MAP kinase
FABP-4	Fatty acid binding protein 4
FAS	Fatty acid synthase
FCS	Foetal calf serum
Glut-1	Solute carrier family 2 (facilitated glucose transporter), member 1
Glut-4	Solute carrier family 2 (facilitated glucose transporter), member 4
HSL	Hormone-sensitive lipase
IBMX	Isobutyl-methylxanthine
IC_50_	Half maximal inhibitory concentration
IL17	Interleukin 17
IL1β	Interleukin 1β
IL6	Interleukin 6
INFG	Inflammatory-related genes
LUC	Luciferase
LXR	Liver X receptor
MCP-1	Monocyte chemoattractant protein 1
M-MLV	Moloney murine leukemia virus
MTT	3-(4,5-dimethylthiazol-2-yl)-2,5-diphenyl tetrazolium
NBBS	New born bovine serum
N-Cor	Nuclear receptor corepressor 1
NIS	Sodium iodide symporter
OD	Optical density
ORO	Red oil O reagent
PAH	Polycyclic Aromatic Hydrocarbons
PAI-1	Plasminogen activator inhibitor 1, or Serpine1
PBS	Phosphate-buffered saline
PBS	Phosphate buffered saline
PCB	Polychlorinated biphenyl
PCBs-DL	PCB-dioxin like
PCR	Polymerase chain reaction
POP	Persistent organic pollutants
PPARγ	Peroxisome proliferator-activated receptor gamma
PXR	Pregnane X receptor
SD	Standard deviation
SDS	Sodium dodecyl sulfate
SMRT	Nuclear receptor corepressor 2
SRC-1	Steroid receptor coactivator-1
SREBP	Sterol regulatory element binding protein
T2D	Type 2 diabetes
T4	Thyroxin
Th17	T helper 17
TNFα	Tumor necrosis factor alpha
XRE	Xenobiotic responsive element

## Appendix A

**Table A1:** Intergroup comparison: the significance of the synergism and antagonism. Significance of differences of gene expression between assays treated with either BaP only, or with BaP + PCBs in “Time 1” and “Time 2”. The intergroup comparisons were obtained with Kruskal–Wallis test followed up by Dunn’s test. The level of significance is * *p* < 0.05; ** *p* < 0.01; *** *p* < 0.001; **** *p* < 0.0001. Genes for which there is no significant difference are indicated in italics. ns: no significant. Genes for which there is no significant difference are indicated in bold.

	**Time 1**			
**Genes**	**BaP vs**. **BaP + PCB118-1 µM**	**BaP vs**. **BaP + PCB118-5 µM**	**PCB118-1 µM vs**. **BaP + PCB118-1 µM**	**PCB118-5 µM vs**. **BaP + PCB118-5 µM**
*CYP1A1*	*****	******	******	******
*PPARγ*	*****	*****	******	******
*Glut-1*	ns	ns	******	******
*Glut-4*	ns	ns	*******	*******
*Lipin-1a*	*****	******	******	******
*Lipin-1b*	ns	ns	******	******
*ATGL*	ns	ns	******	******
*FABP-4*	ns	ns	******	******
*FAS*	*****	*****	******	******
*HSL*	ns	*****	*******	*******
*SREBP*	ns	ns	ns	*****
*TNFα*	ns	ns	*****	*****
*IL-6*	ns	ns	ns	******
*MCP-1*	ns	ns	*******	*******
*CXCL-10*	******	******	******	******
***PAI-1***	ns	ns	ns	ns
*Adiponectin*	*****	*****	********	*******
*Leptin*	ns	ns	******	********
**Genes**	**BaP vs**. **BaP + PCB153-1 µM**	**BaP vs**. **BaP + PCB153-5 µM**	**PCB153-1 µM vs**. **BaP + PCB153-1 µM**	**PCB153-5 µM vs**. **BaP + PCB153-5 µM**
*CYP1A1*	ns	*****	*****	******
*PPARγ*	ns	ns	*******	*******
*Glut-1*	*****	ns	******	*******
*Glut-4*	ns	ns	********	*******
*Lipin-1a*	******	******	******	******
*Lipin-1b*	ns	ns	*******	******
*ATGL*	ns	ns	*******	*******
*FABP-4*	ns	ns	*******	*******
*FAS*	*****	*****	*******	******
*HSL*	ns	ns	*******	*******
*SREBP*	ns	ns	ns	ns
*TNFα*	ns	******	*****	*******
*IL-6*	ns	*****	ns	******
*MCP-1*	*****	ns	*******	*******
*CXCL-10*	*****	*****	******	******
***PAI-1***	ns	ns	ns	ns
*Adiponectin*	*****	*****	*******	*******
*Leptin*	ns	ns	********	********
	**Time 2**			
**Genes**	**BaP vs**. **BaP + PCB118-1 µM**	**BaP vs**. **BaP + PCB118-5 µM**	**PCB118-1 µM vs**. **BaP + PCB118-1 µM**	**PCB118-5 µM vs**. **BaP + PCB118-5 µM**
*CYP1A1*	ns	*****	ns	*****
*PPARγ*	ns	*****	ns	ns
***Glut-1***	ns	ns	ns	ns
*Glut-4*	ns	*****	ns	******
*Lipin-1a*	ns	*****	*****	*****
*Lipin-1b*	ns	*****	*****	*****
***ATGL***	ns	ns	ns	ns
*FABP-4*	*****	ns	ns	ns
*FAS*	*****	ns	ns	ns
*HSL*	*****	ns	*****	ns
***SREBP***	ns	ns	ns	ns
***TNFα***	ns	ns	ns	ns
***IL-6***	ns	ns	ns	ns
***MCP-1***	ns	ns	ns	ns
*CXCL-10*	*****	ns	*****	*****
*PAI-1*	ns	ns	ns	*****
*Adiponectin*	*****	******	ns	******
*Leptin*	ns	ns	*****	******
**Genes**	**BaP vs**. **BaP + PCB153-1 µM**	**BaP vs**. **BaP + PCB153-5 µM**	**PCB153-1 µM vs**. **BaP + PCB153-1 µM**	**PCB153-5 µM vs**. **BaP + PCB153-5 µM**
*CYP1A1*	ns	ns	ns	*****
***PPARγ***	ns	ns	ns	ns
*Glut-1*	ns	ns	*****	*****
*Glut-4*	*****	*****	******	*****
*Lipin-1a*	ns	ns	*****	*****
*Lipin-1b*	ns	ns	*****	*****
***ATGL***	ns	ns	ns	ns
*FABP-4*	ns	ns	ns	*****
*FAS*	ns	*****	ns	*****
*HSL*	ns	*****	ns	*****
***SREBP***	ns	ns	ns	ns
***TNFα***	ns	ns	ns	ns
*IL-6*	ns	*****	ns	ns
*MCP-1*	*****	ns	*****	******
*CXCL-10*	*****	******	*****	******
*PAI-1*	ns	ns	******	******
*Adiponectin*	*****	ns	******	ns
*Leptin*	ns	ns	******	*****

Comments of Figure A2: As anticipated, the basal level of expression of genes related to adipogenesis appeared strongly lower in undifferentiated 3T3-L1 cells than in cells that were differentiated or being differentiated (around ×1/90 for *Glut-4*, 1/60 for *Adiponectin*, 1/25 for *Lipin-1a* and 1/70 for *PPARγ*). The level of Leptin appeared lesser reduced (around 1/2.5).

Concerning *Glut-4*, the low level of expression was slightly increased in response to PCB153 and this increase was abolished by the co-addition of BaP. Regarding *Leptin* and *Adiponectin*, the low level of expression was faintly further reduced by the co-exposure to BaP and PCB118 and 153. Expression of *Lipin-1a* and *PPARγ* was reduced by the co-exposure to BaP and PCB118 and also, for the expression of *PPARγ*, by the mix of BaP and PCB153.

Comparison between the changes of genes expression induced by BaP and PCBs in differentiated versus undifferentiated cells led to the following observations: on the whole, the expression of genes analyzed in undifferentiated cells was slightly down-regulated by the mix of BaP and PCB but these effects were often at the limit of the significance and very faint compared to the down-regulation observed in differentiated cells. In addition, BaP alone induced no effect in undifferentiated cells while a strong decrease of gene expression was induced in differentiated cells.

These data led to the observation that gene expression was much more affected by the studied pollutants in differentiated cells compared to undifferentiated cells. It must be emphasized that the effects of these compounds were strongly enhanced on genes which were induced by the differentiation process.

Comments of Figure A3: The PCB118-induced decrease of *AhR* gene expression was abolished by the co-exposure to BaP, while this compound alone decreased only faintly the expression. A reverse dose-dependent effect is observed with PCB153: indeed, 1 μM of this compound induced an enhancement while 5 μM induced a decrease of the expression. Co-exposure to PCB153 and BaP tends to abolish the PCB153-induced effect.
